# A Comparative Study of Non-Invasive Methods for Fibrosis Assessment in Chronic HCV Infection

**Published:** 2010-06-01

**Authors:** Roxana Şirli, Ioan Sporea, Simona Bota, Alina Popescu, Marioara Cornianu

**Affiliations:** 1 Department of Gastroenterology and Hepatology, University of Medicine and Pharmacy, Timişoara, Romania; 2 Department of Pathology, University of Medicine and Pharmacy, Timişoara, Romania

**Keywords:** Chronic Hepatitis C, Fibrosis, Liver Function Tests

## Abstract

**Background and Aims:**

To compare several non-invasive methods of fibrosis assessment in chronic hepatitis C virus (HCV) infection (platelet count, the APRI score, the Forns score, the Lok score, FIB-4, Transient Elastography [TE]), versus percutaneous liver biopsy (LB).

**Methods:**

Our study included 150 patients with chronic HCV infection in which LB, liver stiffness measurement (LSM) by means of TE and biological tests needed for calculating the scores (according to the classic formulas) were performed in the same session.

**Results:**

The best test for predicting significant fibrosis (F = 2 Metavir) was LSM with AUROC-0.773, followed by APRI (AUROC-0.763), Forns (AUROC-0.744), platelet count (AUROC-0.732), Lok (AUROC-0.701) and FIB-4 (AUROC-0.669), but the differences were not statistically significant (P > 0.05). For excluding cirrhosis, all the tests had excellent NPV (>97%). The best test for predicting cirrhosis was LSM (AUROC-0.979), significantly better than platelet count (AUROC- 0.899, P = 0.022) and than FIB-4 (AUROC-0.839, P = 0.042), otherwise the differences were not statistically significant (P > 0.05). All of the non-invasive tests were statistically significantly correlated (P < 0.0001) to the severity of fibrosis: APRI r=0.570; Forns r=0.540; Lok r=0.4843; FIB-4 r=0.4171; platelet count r=-0.4842.

**Conclusions:**

LSM by means of TE seems to be more sensitive than APRI, Forns, Lok and FIB-4 scores and than platelet count for the prediction of significant fibrosis, but the differences are not statistically significant. The APRI score and Forns scores correctly identified most (71%) of the patients having, or not having, significant fibrosis. LSM was the best method for predicting cirrhosis, but all the evaluated tests had excellent predictive value (AUROCs 0.839-0.979).

## Introduction

Liver biopsy (LB) plays a key role in the diagnosis and monitoring of diffuse chronic hepatitis, especially for its staging [1][2][3][4]. The prognosis and management of a patient with chronic hepatitis C virus (HCV) infection depend very much on the severity of liver fibrosis [4], which can be assessed by several methods: LB, considered the “gold standard”; serological markers (FibroTest being the most frequently used); and elastographic methods: noninvasive techniques based on liver tissue elasticity.

Although considered the “gold standard”, LB is not a perfect method; there are a number of problems related to the diagnosis of cirrhosis [5]: the inequality of fibrosis in the two liver lobes in paired LB [6]; also inter-and intra-observer variability in the evaluation of specimens obtained by LB [5][7]. In addition, LB is an invasive maneuver, (with a risk of complications, even if it is low) causing discomfort for the patients [8][9][10][11].

For these reasons, non-invasive methods of assessing the severity of fibrosis which may someday completely replace LB, are constantly being searched for. Among the non-invasive tests, the best results were obtained with liver stiffness measurement (LSM) by means of transient elastography (TE) (FibroScan®), and with FibroTest-ActiTest® (Biopredictive, Labcorp) [12] and Fibrospect II® (Prometheus) [13]. All these non-invasive methods are expensive and/or require equipment that is not widely available; therefore simpler, cheaper methods for the prediction of hepatic fibrosis were sought for.

The aim of this study was to evaluate several simple serological tests for the prediction of fibrosis in chronic HCV infection: number of platelets, the APRI test, the Forns score, the Lok score and the FIB-4 score; as compared to LSM by TE and to the current “gold standard”: the LB.

## Materials and Methods

### Patients

The study was retrospective and included 150 cases of chronic HCV infection admitted to the Department of Gastroenterology and Hepatology, Timisoara during January-December 2008. In all of these patients, in the same session, liver stiffness (LS) was evaluated by means of TE (FibroScan®); LB was performed in order to assess the stage of fibrosis; and the biological samples needed to calculate the scores were collected. Also, all of the patients were evaluated by abdominal ultrasound to exclude those with ascites. Patients with other causes of chronic hepatitis (Hepatitis B virus [HBV] infection, chronic alcohol abuse, cholestatic chronic hepatitis, nonalcoholic steatohepatitis, autoimmune chronic hepatitis, haemochromatosis, Wilson’s disease) were excluded from our study, based on negative hepatitis B surface antigen (HBsAg), negative history of alcohol abuse, no cholestasis on biological tests, no or mild steatosis on abdominal ultrasound and LB, normal iron load, normal ceruloplasmin, negative markers of autoimmune hepatitis or primary biliary cirrhosis, and no signs of biliary obstruction on abdominal ultrasound.

Informed consent was obtained from each patient included in the study and the study protocol was approved by the local ethical committee.

### LSM by means of TE

TE was performed on all of the 150 patients with a FibroScan® device (EchoSens® - Paris, France) by 3 experienced physicians. In each patient, 10 valid measurements were taken, after which a median value of LS was calculated, measured in kiloPascals (kPa). Only patients in which LSMs had a success rate of at least 60%, with an interquartile range (IQR) of <30%, were included in our study. The success rate was calculated as the ratio of the number of successful acquisitions to the total number of acquisitions. IQR is the difference between the 75th percentile and the 25th percentile, essentially the range of the middle 50% of the data.

### Liver biopsy

Echoassisted LB was performed on all 150 patients using Menghini-type modified needles, 1.4 and 1.6 mm in diameter. Only LB fragments of at least 2 cm, including at least 8 portal tracts were considered adequate for pathological interpretation and were included in the study. All the LBs were assessed according to the Metavir score by a senior pathologist. Fibrosis was staged on a 0–4 scale: F0 - no fibrosis; F1 - portal fibrosis without septa; F2 - portal fibrosis and a few septa extending into lobules; F3 - numerous septa extending to adjacent portal tracts or terminal hepatic venules and F4 – cirrhosis.

### Biological tests

Biological tests were performed on blood collected by venous puncture in a single laboratory at the County Emergency Hospital in Timisoara. All biological tests are routinely evaluated in this laboratory: AST - normal values 5-34 U/l; ALT: normal values 10-35 U/l; GGTP: normal values 12-64 U/l; platelets: normal values 150,000-450,000/mm3; INR: normal values 0.88-1.10; cholesterol: normal values <200 mg%.

### Serological tests for assessing fibrosis

We evaluated the following serological tests for the assessment of fibrosis:

a)The platelet count

b)The APRI score (AST/Platelet ratio index) is calculated according to the formula: APRI = [(AST/ULN) x 100]/platelet count 10(9)/L where ULN = the upper limit of normal.

c)The Forns score, calculated according to the formula: Forns score = 7.811 - 3.131 x ln [platelet count (10(9)/L)] + + 0.781 x ln[(GGTP (IU/L)] + 3.467 x ln [age(years)] – 0.014 [cholesterol (mg/dL)]

d) The Lok score, calculated according to the formula: Log odds = - 5.56 – 0.0089 x platelet count (10(3)/mm(3)) + 1.26 x (AST/ALT) + 5.27 x INR Lok = [exp (logodds)]/[1 + exp (logodds)]

e)The FIB-4 score, calculated according to the formula: FIB-4 = [age (years) x AST (IU/L)]/[platelet count (10(9)/L) x ALT (IU/L)1/2].

### Statistical analysis

Data obtained from the patients were collected in a Microsoft Excel file. For a statistical study of quantitative variables, the mean and standard deviations were calculated. The diagnostic performances of LSMs and of the serologic tests were assessed by using the area under the receiver operating curve (AUROC), the most widely used indicator of accuracy (when the AUROC value is approaching 1, the accuracy is high). ROC curves were thus built for the detection of significant fibrosis (F = 2 Metavir) and cirrhosis (F = 4 Metavir). Optimal cut-off values were chosen to maximize the sum of sensitivity (Se) and specificity (Sp). Positive predictive values (PPV), negative predictive values (NPV), positive likelihood ratios (+LR) and negative likelihood ratios (-LR) were also assessed. We calculated 95% confidence intervals (CI) of the AUROC curves to compare their predictive values. We also evaluated the correlation between the non-invasive tests and the histological severity of fibrosis. Statistical analysis was performed using Microsoft Excel, GraphPad Prism and MedCalc programs.

## Results

### Patients

**Figure 1:**
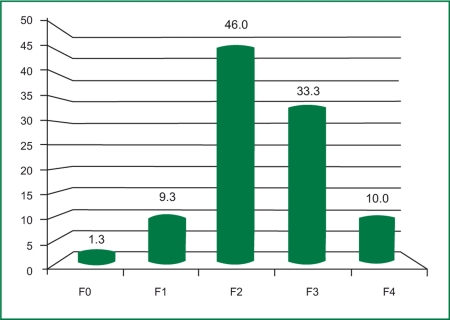
Severity of fibrosis in the studied group.

The study group included 150 patients: 102 women (68%) and 48 men (32%). The mean age of the patients was 50.4±10.3 years, ranging from 18 to 65 years. In the studied group, 1.3% (2 patients) had no fibrosis (F0); 9.3% (14 patients) had stage 1 fibrosis (F1); 46% (69 patients) had stage 2 fibrosis (F2); 33.3% (50 patients) had stage 3 fibrosis (F3); and 10% (15 patients) had cirrhosis (F4), according to the Metavir score (Fig. 1).

LS measurements were obtained for only 144 out of the 150 patients (96%).

### Correlation with the histological severity of fibrosis

All of the non-invasive tests were statistically significantly correlated to the severity of fibrosis, either directly (APRI score, Forns, Lok and FIB-4 scores); or inversely (platelet count), as seen in Table 1. The strongest correlation was found for the APRI score (r = 0.570) and LSM ( r= 0.5694).

**Table1:** Correlation between the evaluated tests and the severity of fibrosis.

**Test**	**Range**	**Correlation score r**	**95% CI**	**P-value**
Platelet count	89,000-399,000/mm^3^	-0.4842	-0.6012 to -0.3470	<0.0001
APRI score	0.16 - 4.3	0.570	0.4483-0.6726	<0.0001
Forns score	0.74 - 10.04	0.540	0.4118-0.6473	<0.0001
Lok score	0.02 - 0.6	0.4843	0.3466-0.6016	<0.0001
FIB-4 score	0.22 - 6.63	0.4171	0.2707-0.5446	<0.0001
LSM	3.1 - 26.3kPa	0.5694	0.4436-0.6732	<0.0001

### Prediction of significant fibrosis (F ≥ 2 Metavir)

The results of the statistical analysis of the predictive value of the non-invasive tests for the presence of significant fibrosis are summarized in Table 2. All the tests had very good positive predictive values (PPV) (95-100%), good specificity (Sp), but their sensitivity (Se) and negative predictive value (NPV) were low.

**Figure 2:**
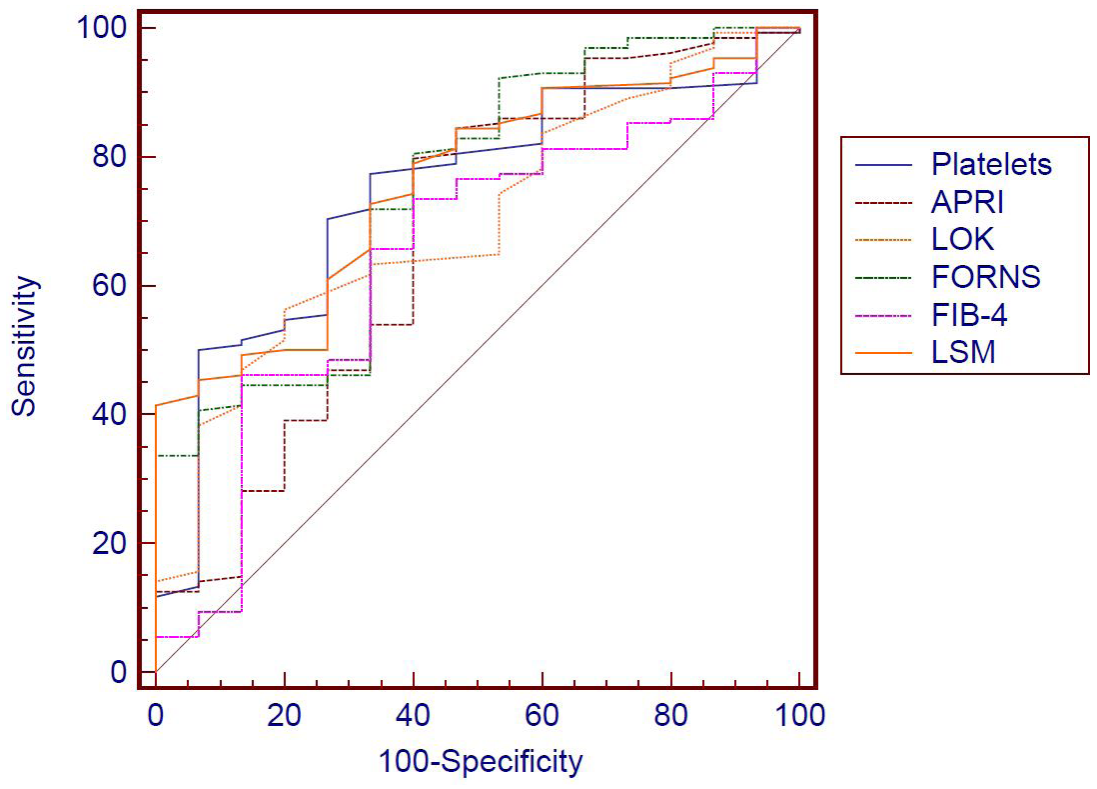
AUROC curves for the prediction of significant fibrosis (F ≥ 2 Metavir) of the evaluated non-invasive tests.

We compared the AUROC curves of the 6 non-invasive tests, built for the prediction of significant fibrosis (F = 2 Metavir), by using the 95% CI and the standard error of the mean (SE), and found that although the LSM, done by means of TE and the APRI score, seem to have a somewhat better predictive value (larger AUROC); the differences are not statistically significant, both between the two mentioned tests, and in the other tests evaluated (P > 0.05) (Fig. 2).

**Table2:** Predictive value of the non-invasive tests for the presence of significant fibrosis (F ≥ 2 Metavir).

**Test**	**Cut-off**	**AUROC**	**SE**	**95% CI**	**P-value**	**Se (%)**	**Sp (%)**	**PPV (%)**	**NPV (%)**	**+ LR**	**- LR**
Platelet count (/mm^3^)	176,000	0.732	0.0746	0.650-0.798	<0.0001	37.3	100	100	16	-	0.63
APRI	0.52	0.766	0.0539	0.688-0.833	<0.0001	70	81	97	24.5	2.81	0.40
Forns	4.57	0.748	0.0566	0.668-0.816	<0.0001	71.6	68.5	95	24	2.29	0.41
Lok	0.17	0.701	0.0627	0.619-0.774	0.0009	57.5	81.2	96.2	18.6	3.06	0.52
FIB-4	2.1365	0.686	0.0644	0.603-0.761	0.0085	35.8	100	100	15.7	-	0.64
LSM (kPa)	6.8	0.773	0.0553	0.678-0.824	<0.0001	60.5	73.4	95	17.7	3.53	0.64

### Prediction of cirrhosis (F = 4 Metavir)

The results of statistical analysis on the predictive value of the non-invasive tests for the presence of cirrhosis are summarized in Table 3. All of the tests have very good NPV (>97%), also good Se (>80%) and Sp, thus allowing the exclusion of cirrhosis.

**Figure 3:**
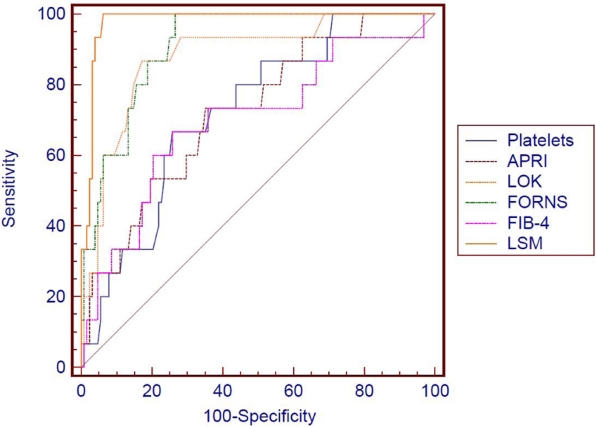
AUROC curves for the prediction of cirrhosis (F = 4 Metavir) of the evaluated non-invasive tests.

We compared the AUROC curves of the 6 non-invasive tests, built for the prediction of cirrhosis (F = 4 Metavir), by using the 95% CI and the SE, and found out that LSM by TE is the best method for predicting cirrhosis, significantly better than platelet count (P = 0.022) and than FIB-4 (P = 0.042); otherwise the differences were not statistically significant (P > 0.05) (Fig. 3).

**Table3:** Predictive value of the non-invasive tests for the presence of cirrhosis (F = 4 Metavir).

**Test**	**Cut-off**	**AUROC**	**SE**	**95% CI**	**P-value**	**Se (%)**	**Sp (%)**	**PPV (%)**	**NPV (%)**	**+ LR**	**- LR**
Platelets (/mm^3^)	155,000	0.899	0.0301	0.838-0.943	<0.0001	86.7	83.7	37.1	98.3	5.32	0.16
APRI	1.38	0.909	0.0519	0.850-0.951	<0.0001	93.3	83	37.8	99	5.48	0.08
Forns	5.93	0.911	0.0514	0.852-0.952	<0.0001	100	74	30	100	3.86	0
Lok	0.26	0.873	0.0596	0.808-0.923	<0.0001	86.7	82.2	35.1	98.2	4.87	0.16
FIB-4	2.3122	0.842	0.0649	0.772-0.898	<0.0001	80	77.8	28.6	97.2	3.6	0.26
LSM (kPa)	13.3	0.979	0.0262	0.850-0.951	<0.0001	93.3	96.1	73.7	99.2	24.08	0.07

## Discussion

Non-invasive tests for the assessment of the severity of hepatic fibrosis are gaining ever more ground among hepatologists, who are now put in the difficult position of choosing which one to use, taking into account that, at this time more than 20 biochemical tests are available, not to mention elastographic methods [14].

All of these non-invasive tests have good PPV for the diagnosis of minimal or absent fibrosis and severe fibrosis, respectively [15][16][17][18]; but their practical usefulness is limited by such factors as the need to standardize methods of determining the parameters taken into account for the test in question (FibroTest), the high cost of the device (FibroScan), and the possibility of false positive and false negative results. Platelet count. Starting from the fact that thrombocytopenia is a recognized complication of liver cirrhosis, usually secondary to hypersplenism, an attempt was made to establish its predictive value for the severity of fibrosis in chronic HCV infection. Two large studies published in 2005 showed that a cut-off of 150,000/mm3 in the platelet count had a PPV of 90% for the presence of cirrhosis. Also, a platelet count higher than 150,000/mm3 had a NPV of 90% for cirrhosis [19],[20].

In our study we found that the platelet count,indirectly, was statistically significantly correlated with the severity of fibrosis: r = - 0.4842 (95%CI: -0.6012 to -0.3470), P < 0.0001. Also, a platelet count smaller than the cut-off value of 176.000/mm3 proved to be a predictor with a high Sp-100% of significant fibrosis (F=2 Metavir) but with a low Se (37.3%), with 100% PPV, but with a low NPV-16%. Using this cut-off value, 68% (102/150) of the patients were correctly classified as either having, or not having, significant fibrosis.

Regarding cirrhosis, a platelet count higher than 155.000/mm3 proved to be a good predictor for the exclusion of cirrhosis, with 83.7% Sp, 86.7% Se and 98.3% NPV, also with 5.32 +LR. For this cut-off, 91% (137/150) of the patients were correctly classified as having or not having cirrhosis.

The APRI score is not an expensive test, and thus within reach of any clinician. Various studies report quite different performance scores for the staging of fibrosis in HCV chronic hepatitis: 41-91% Se, 47-95% Sp and 60-82.7% accuracy for predicting significant fibrosis (F = 2 Metavir); 38.4-65.8% Se, 86.7-93% Sp and 60-88% diagnostic accuracy for predicting cirrhosis [21][22][23].

A meta-analysis [24] from 2007 proved that for a cut-off value of 0.5, the APRI score had 81% Se and 50% Sp for predicting significant fibrosis (F = 2 Metavir) and that for a cut-off value of 1, the Se and Sp for predicting cirrhosis were 76% and 71%.

In our study, for a cut-off value of 0.52, the APRI score had 70% Se and 81% Sp for predicting significant fibrosis (F = 2 Metavir), with 97% PPV and 24.5% NPV and 2.81 +LR. For a cut-off value of 1.38, the APRI score had 93.3% Se, 83% Sp and 5.48 +LR for the diagnosis of cirrhosis. For the cut-off value of 0.5 proposed by Shaheen meta-analysis [24], the APRI was slightly more sensitive (73% vs. 70%), but not as specific (75% vs. 80%) for predicting significant fibrosis (F = 2 Metavir). For cirrhosis prediction, at the cut-off value of 1 [24] Se remained at 93.3%, but the Sp decreased significantly to 69% (vs. 83%).

For a cut-off value of 0.52, 71% (107/150) of the patients were correctly classified as having or not having significant fibrosis, and for a cut-off value of 1.38, 82% (123/150) of patients were correctly classified as having or not having cirrhosis. For a cut-off value of 1, as recommended by Shaheen, 70.6% (106/150) were correctly classified.

The Forns score is a simple score that takes into account the patient’s age, GGTP and serum cholesterol levels and the platelet count. The prediction accuracy for significant fibrosis in chronic HCV infection was reported to be between 50 and 85% [23],[25]. The value of this test is lower than of the FibroTest in the diagnosis of significant fibrosis [26],[27]. Also, the Forns score does not provide information on cirrhosis, leaving almost half of the cases unclassified [28].

In our study, for a cut-off value of 4.57, the Forns score had 71.6% Se and 68.5% Sp in discriminating significant fibrosis (F = 2 Metavir), with 95% PPV, 24% NPV and 2.29 +LR. A Forns score higher than 4.2 (low cut-off value recommended by the authors of the score) had 76% Se, 56% Sp, 93.6% PPV and 22 % NPV for predicting significant fibrosis, meaning that the method is able to identify patients with significant fibrosis, but is not sensitive enough. For a cut-off value of 4.57, 71% (107/150) of the patients were correctly classified as having or not having significant fibrosis.

The Lok score was proposed by the group led by Ann Lok during the Halt-C trial [20]. According to the authors, for a cut-off value smaller than 0.2 to exclude cirrhosis, only 7.8% of patients had been wrongly classified (98% Se, 53% Sp, 27% PPV and 99% NPV), and for values higher than 0.5 to confirm cirrhosis, only 14.8% of patients had been wrongly classified (40% Se, 99% Sp, 84% PPV and 90% NPV).

In our study, for a cut-off value of 0.26 (which maximizes the sum of Se and Sp), the Lok score had 86.7% Se and 82.2% Sp for cirrhosis discrimination (F = 4 Metavir), with 35.1% PPV, 98.2% NPV and 4.87 +LR. At values lower than a cut-off of 0.2, the Lok score accurately excluded cirrhosis (99% NPV, 93% Se, and 66% Sp). For values greater than a cut-off of 0.5, Lok score accurately predicted cirrhosis (91.7% PPV, 98.5% Sp, 60% NPV and 20% Se). For a cut-off value of 0.5, 91.3% (137/150) of patients were correctly classified as having or not having cirrhosis and for a cut-off value of 0.2, 69.3% (104/150) of patients were correctly classified.

The FIB-4 score was originally developed for human immunodeficiency virus (HIV)-HCV coinfection, but was confirmed also for HCV infection, with performances similar to the FibroTest [29],[30] for the diagnosis of severe fibrosis (F3 and F4), with AUROC 0.85 (95%CI: 0.82-0.89). For values lower than a cut-off of 1.45, FIB-4 excluded severe fibrosis with a good NPV (94.7%), with 74% Se and 80% Sp. For values higher than a cut-off of 3.25, FIB-4 confirmed severe fibrosis with 82% PPV, 37.6% Se and 98% Sp. For predicting cirrhosis, FIB-4 had an AUROC of 0.91.

In our study, for a cut-off value of 2.1365, the FIB-4 score had 35.8% Se and 100% Sp in discriminating significant fibrosis (F = 2 Metavir), with 100% PPV and 15.7% NPV, 42% of the patients (64/150) being correctly classified as having or not having significant fibrosis. Also, for a cut-off of 2.3122, FIB-4 had 80% Se and 77.8% Sp in cirrhosis discrimination, with 28.6% PPV, 97.2% NPV and 3.6 +LR, 98% (147/150) of the patients being correctly classified as having or not having liver cirrhosis.

LSM by means of TE is a method which has been proved useful for predicting significant fibrosis (cut-off values 7.1-8.7kPa) and cirrhosis (cut-off values 12.5-14.5kPa) [17],[31]. A recent meta-analysis [32] has confirmed the excellent performance of TE for the diagnosis of cirrhosis: mean AUROC - 0.94 (95% CI: 0.93-0.95). As to its predictive value for significant fibrosis (F=2 Metavir), the mean AUROC was 0.85 (95% CI: 0.81-0.87), the suggested cut-off being 7.65 kPa.

In our study, for a cut-off value of 6.8 kPa, LS had 60.5% Se, 73.4% Sp for discriminating significant fibrosis (F = 2 Metavir), with 95% PPV, 17.7% NPV and 3.53 +LR, 66% (95/144) of the patients being correctly classified as having or not having significant fibrosis. For a cut-off value of 13.3 kPa, LS had 93.3% Se, 96.1% Sp for the diagnosis of cirrhosis, with 73.7% PPV, 99.2% NPV and 24.08 +LR, 96.5% (137/144) of the patients being correctly classified as having or not having cirrhosis.

When we compared the AUROC curves built to assess the predictive value for significant fibrosis of the evaluated non-invasive tests, we found no statistically significant differences, even if LSM (AUROC 0.773) and the APRI score (AUROC 0.766) seem to be better (with a larger AUROC) (P > 0.05). The weakest predictive values were found for the Lok score (AUROC 0.701) and the FIB-4 score (AUROC 0.686), both scores initially developed for the prediction of cirrhosis (Lok), or severe fibrosis (FIB-4). Considering LR according to Jaeschke et al. [33], when values higher than the cut-off ones for APRI score, Forns score, Lok score and LSM were obtained, the probability of patients having significant fibrosis (F = 2) was significantly increased (+LR: 2.81, 2.29, 3.06 and 3.53 respectively), while smaller values then the calculated cut-offs, for the all the investigated testes, had poor predictive value for excluding significant fibrosis (-LR ranging from 0.4 to 0.64).

As for cirrhosis, all the tests had excellent predictive values, with AUROCs of 0.839-0.979. LSM was the best method for predicting cirrhosis, significantly better than platelet count (P = 0.022) and than FIB-4 (P = 0.042), otherwise the differences were not statistically significant (P > 0.05). In terms of LRs, LSM was the best test to predict or exclude cirrhosis (+LR 24.08 and –LR 0.07). All the tests had very good –LRs (ranging from 0 to 0.26), meaning that a negative test was very good for excluding cirrhosis.

One limitation of our study is the fact that it is a retrospective one; and another is the small number of cirrhotic patients. Also, several published studies stated the influence of inflammation [34],[35], steatosis [35] and ALT flares [36] on LSMs in patients with chronic HCV infection. These factors were not taken into consideration in our study.

## Conclusions

LSM by means of TE seems to be more sensitive than APRI, Forns, Lok and FIB-4 scores and platelet count for the prediction of significant fibrosis, but the differences are not statistically significant. APRI score and Forns scores correctly identified most (71%) of the patients as having or not having significant fibrosis. LSM was the best method for predicting cirrhosis, but all the evaluated tests had excellent predictive value (AUROCs 0.839-0.979).
